# Electrochemical Polymerisation of Glutamic Acid on the Surface of Graphene Paste Electrode for the Detection and Quantification of Rutin in Food and Medicinal Samples

**DOI:** 10.3390/diagnostics12123113

**Published:** 2022-12-09

**Authors:** Balliamada M. Amrutha, Jamballi G. Manjunatha, Hareesha Nagarajappa, Ammar M. Tighezza, Munirah D. Albaqami, Mika Sillanpää

**Affiliations:** 1 Department of Chemistry, FMKMC College, Mangalore University Constituent College, Madikeri 571201, Karnataka, India; 2 Department of Chemistry, College of Science, King Saud University, Riyadh 11451, Saudi Arabia; 3 Department of Biological and Chemical Engineering, Aarhus University, Nørrebrogade 44, 8000 Aarhus, Denmark

**Keywords:** voltammetry, rutin, graphene, glutamic acid, caffeine, electrochemical analysis

## Abstract

Rutin (RU) is one of the best-known natural antioxidants with various physiological functions in the human body and other plant species. In this work, an efficient voltammetric sensor to detect RU in food samples was explicated using a poly (glutamic acid)-modified graphene paste electrode (PGAMGPE). In order to detect RU, the proposed sensor diminishes material resistance and overpotential while increasing kinetic rate, peak currents, and material conductance. Using differential pulse voltammetry (DPV) and cyclic voltammetry (CV), the analysing efficiency of a PGAMGPE and a Bare graphene paste electrode (BGPE) was evaluated in 0.2 M phosphate buffer (PB) at an ideal pH of 6.5. in a potential window of −0.25 V to 0.6 V. Electrochemical impedance spectroscopy (EIS) was used to analyse the prepared electrode materials’ conductivity, charge transfer resistance, and the kinetics of electron transport. Field emission scanning electron microscopy (FE-SEM) images were considered to compare the exterior morphology of the PGAMGPE and the BGPE. It was discovered that the PGAMGPE and the BGPE have electroactive surfaces of 0.062 cm^2^ and 0.04 cm^2^, respectively. It was determined that two protons and two electrons participated in the redox process. The resultant limit of detection (LOD) was found to be 0.04 µM and 0.06 µM, respectively, using DPV and CV methods. In spite of common interferents such as metal ions and chemical species, the developed sensor’s selectivity for RU detection was impressive. For the simultaneous analysis of RU in the presence of caffeine (CF), the PGAMGPE affords a good electrochemical nature for RU with good selectivity. Due to the good stability, repeatability, reproducibility, and ease of use of the present RU sensor, it is useful for real sample analysis such as food and medicinal samples with recovery ranging from 94 to 100%.

## 1. Introduction

A polyphenolic bioflavonoid RU, also known as sophorin, rutoside, and quercetin-3-rutinoside is mostly taken from citrus fruits, grapes, berries, tea, peaches, and certain other naturally occurring supplies. Chemically, it is a glycoside composed by the flavonolic quercetin and disaccharide rutinose [1]. Several plant species naturally produce RU, and it can be found in buckwheat seed, fruit rinds, and other plant parts, particularly citrus fruits (orange, grapefruit, and lemon). The oxidising species OH radical, superoxide radical, and peroxyl radical can all be effectively scavenged by RU. As a result, it exhibits a range of pharmacological activity, including anti-inflammatory, antiallergic, and vasoactive properties as well as anticancer, antibacterial, antiviral, and antiprotozoal ones. RU is also said to have various therapeutic effects such as hypolipidemia, anticarcinogenic properties, and antidiabetic properties. RU has a benefit over flavonoids such as myricetin, quercetagenin, and others that can occasionally act as pro-oxidants and promote the creation of oxygen radicals. In comparison to glycones, whose use as medicinal agents is prohibited due to their mutagenic and cytotoxic action, RU has additional advantages [2]. RU is a lipophilic substance that is soluble in pyridine, methanol, and ethanol [3]. As a result, it may be worthwhile to think of it as a potentially useful molecule that is both harmless and inert. Hence, it is essential to analyse RU in foodstuff, biological, and pharmaceutical samples with a methodology that is more straightforward, economical, eco-friendly, and adaptive.

Various analytical methods such as high-performance thin-layer chromatography [4], capillary electrophoresis [5], ultraviolet–visible spectrophotometry [6], high-performance liquid chromatography with ultraviolet detection [7], chromatographic fingerprint analysis [8], chemiluminescence [9], capillary zone electrophoresis [10], high-performance liquid chromatography mass spectrometry [11], etc., have been used for the sensitive detection of RU. However, all these analytical procedures are lengthy, challenging to handle with expensive instrumentation, require difficult pretreatment procedures, provide high LOD, low sensitivity, and it is difficult to analyse the target molecule in the presence of interferents [12].

Fabrication of an electrochemically sensitive working electrode with appropriate surface modification is necessary for the detection of RU. During the past few decades, graphene and other carbon-based substances have been widely employed for the investigation of various significant electrochemical compounds [13]. Graphene is well known for having a variety of interesting electrochemical characteristics for sensing. These intriguing nanoparticles are composed of two-dimensional short stacks of graphene sheets which are structured like platelets. They are reasonably priced, have a larger potential interface, active surface area, good thermal and electrical conductivity, rapid electron transfer rate, and influential mechanical and chemical stability [14]. Due to the swift method, flexibility of its use, relatively inexpensive nature and ecological benefits, electropolymerisation is one of the most popular techniques for modifying the electrode surface. During this process, an electrochemical reaction takes place at the interface of the electrode which results in effortlessly adhering a conductive polymer film of desired thickness under different conditions of deposition. The dispersed monomers in the electrochemical solution will be reduced to polymers by electrochemical oxidation of monomers, and they form a film on the exterior of the electrode with large number of active sites [15,16,17].

Polyglutamic acid (PGA) is, however, one biomaterial with a variety of biological functions and potential for use in fundamental and functional research. According to the literature, PGA offers both conducting bridges and speeds up electron transport. It consists of many glutamic acid (GA) units connected by γ-peptide links, and the α-carboxyl groups are free to interact with a variety of substances [18]. A GA molecule that combines an amino and a carboxyl group has a variety of special characteristics. It may easily create covalent connections and electrostatic interactions with particular molecules. Additionally, PGA is regarded as a biofriendly polymer due to its adaptable structure and superior biocompatibility. The PGA film which exhibits outstanding qualities in electrochemical applications with different chain length may also be broken down by bacteria. Since it is so simple to prepare, PGA is frequently utilised to modify the electrode. It will increase its adsorption capability when mixed with other materials, particularly when combined with graphene [19].

A modest and responsive PGAMGPE was developed on the basis of electropolymerisation of GA at GPE for RU detection in food and medicinal samples using the CV and DPV methods. This electrochemical sensor (PGAMGPE) presents lower LOD than other complex electrochemical sensors and biosensors (further difficulties during the preparation of electrodes) with precise and responsive RU redox peaks. Moreover, the interfering component’s effect on RU was analysed at PGAMGPE with inspiring results. The sensor (PGAMGPE) is also biodegradable and just requires a modest amount of inexpensive electrode material. The present PGAMGPE has not been applied as a catalyst for the electrocatalytic analysis of RU in any earlier published research data.

## 2. Experimental Procedure

### 2.1. Chemicals and Reagents 

Tokyo Chemical Industry (Japan) supplied RU and graphene with dimensions of 6.0–8.0 nm thick and 15 µm broad. From Molychem (India), silicon oil with a purity of 90% was purchased. Dimethyl sulfoxide (DMSO) was purchased from Sigma Aldrich (India). We bought Na_2_HPO_4_·2H_2_O, NaH_2_PO_4_·H_2_O and K_4_[Fe(CN)_6_] from Himedia chemicals (India). KCl with a purity of 99.5% was obtained from Nice chemicals (India). All the chemicals used were all analytical reagent quality and were used as is. By dissolving them in a specified quantity of solvent, all the necessary chemical solutions with known concentrations were made.

### 2.2. Instrumentation

The electrochemical response of RU was analysed using CHI-6038E from USA. A three-electrode assembly was attached to this electrochemical analyser, where the reference electrode was a saturated calomel electrode (SCE), platinum wire was the auxiliary electrode, and BGPE or PGAMGPE of 3 mm module was the working electrode. The EQ-610 pH device was used to prepare PB with varied pH values ranging from 5.5 to 8.0. The FE-SEM was performed using a ZEISS apparatus at Mangalore University, India.

### 2.3. Fabrication of Working Electrodes

In order to create a homogenous paste, graphene powder and silicone oil were correctly combined in a ratio of 60:40 to obtain BGPE. This homogeneous paste was inserted into the 3 mm gap of the polytetrafluoroethylene tube and its surface was scrubbed by a soft tissue paper to attain a consistently smooth surface on the sensor. The prepared sensor’s exterior was rinsed with double distilled water to get rid of the impurities. The PGAMGPE was fabricated using the electrochemical polymerisation procedure. Ten CV cycles were used with a potential gap of 0.2 V to 1.5 V to electrochemically polymerise 1.0 mM GA in 0.2 M PB on the surface of the BGPE. The exterior of the prepared sensors was then gently cleaned with distilled water to remove the GA molecules that had not been adsorbed.

### 2.4. Preparation of RU Containing Real Sample Solutions

Fresh lime juice was extracted from fresh limes, which was then continuously stirred with DMSO. After that, lime juice and its rind were separated by centrifugation. Whatman filter paper was used to filter the resulting solution. For further examination, the resulting solution of lime juice was diluted with 0.2 M PB. The green tea sachets were submerged for one hour in a container containing DMSO solvent. To obtain a clean solution of green tea, the resultant tea solution was then filtered using filter paper. For further investigation, the resulting stock solution was diluted with PB of pH 6.5. The tablets containing RU were purchased from a neighbourhood pharmacy. The tablet was thoroughly ground into powder, and the resulting powder was then combined with a known quantity of DMSO while being constantly stirred. A clear solution was obtained after filtering the obtained mixture, and the resultant tablet solution was diluted with M PB for additional examination.

## 3. Results and Discussions

### 3.1. FE-SEM Characterisation of BGPE and PGAMGPE 

**Figure 1:**
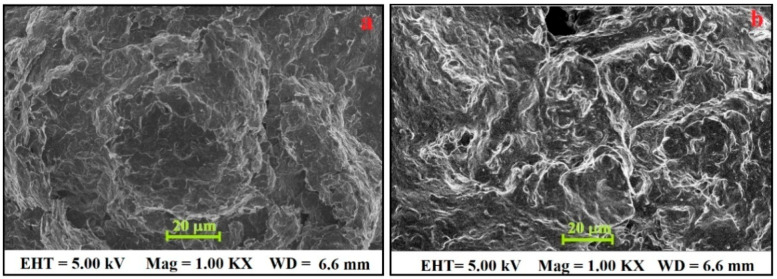
(**a**) FE-SEM picture of BGPE. (**b**) FE-SEM picture of PGAMGPE.

The prepared working electrode surfaces were initially investigated by FE-SEM. Figure 1a displays the typical FE-SEM depiction of the BGPE which reveals a flake-shaped sheet structure with flat morphology indicating the presence of graphene nanoplates with topological size. The BGPE exhibits a layered heterogeneous structure with crumpled and ridged appearance. Figure 1b shows the deposition of a thin layer of PGA film which displays a large surface area for the adsorption of target species indicating that they are finely blended [20]. 

### 3.2. Electrochemical Impedance Studies and Electroactive Surface Area Calculation

**Figure 2:**
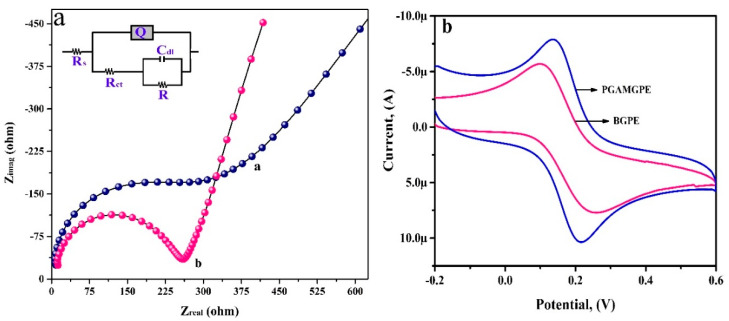
(**a**) Nyquist plot for BGPE (curve a) and PGAMGPE (curve b). (**b**) CVs representing K_4_ [Fe(CN)_6_] (1 mM) on BGPE and PGAMGPE in 0.1 M KCl at the scan rate of 0.1 V/s.

EIS is widely used to evaluate the charge transfer capability of working electrodes. The Nyquist plots of the BGPE and the PGAMGPE were recorded in KCl (0.1 M) with 1.0 mM K_4_[Fe(CN)_6_]. The resistance to electrochemical reaction at the working electrode surface is directly reflected by the semicircle diameter in Nyquist plots as shown in Figure 2a. Due to the weak conductivity of the BGPE, the semicircular portion is significantly greater than the PGAMGPE, and as a result, the R_ct_ value of the BGPE is significantly higher than the PGAMGPE [21,22]. The fitting results showed that the R_ct_ values of the BGPE and the PGAMGPE were 387.1 Ω and 262 Ω, respectively. The R_ct_ values manifest that the polymer film of GA has strong electrical conductivity and the modified electrode has a faster rate of electron transfer. The corresponding circuit was also fitted using the Nyquist plot and had the resulting parameters: solution resistance (R_s_), capacitance (C), R_ct_, and constant phase element (CPE).

Analysis of the electrochemical characteristics of electrodes using CV is a reliable method. Using 1.0 mM K_4_ [Fe (CN)_6_] as a standard redox system with 0.1 M KCl as the supporting electrolyte, the voltammetric performance of the PGAMGPE and the BGPE was examined. The cyclic voltammograms (CVs) in Figure 2b demonstrate that the PGAMGPE has improved current sensitivity in comparison to the BGPE. In the standard redox system, the cathodic and anodic signals had peak to peak separations (ΔEp) of 0.0838 V and 0.1594 V for the PGAMGPE and the BGPE, respectively. The lowered ΔEp value of the modified electrode proves that the polymer coating on the bare electrode’s surface speeds up the rate of electron transport [23].

**Figure 3:**
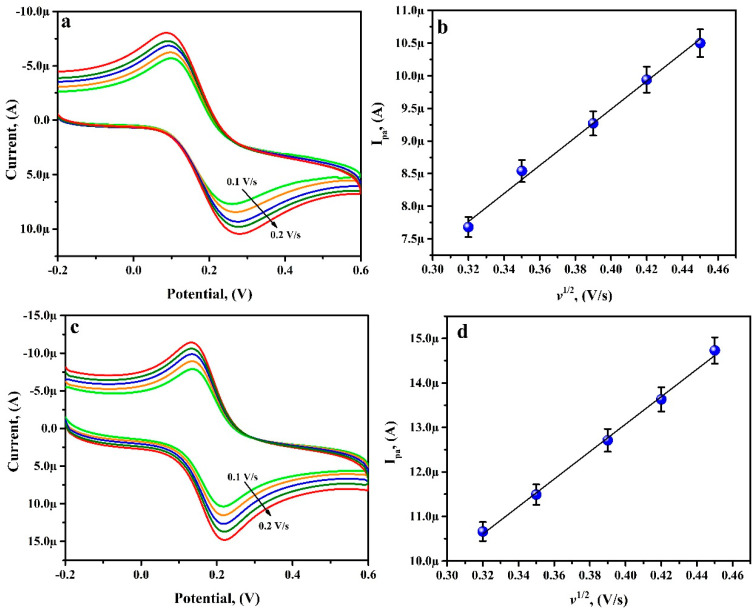
(**a**) CVs of K_4_[Fe(CN)_6_] (1.0 mM) in KCl (0.1 M) on the BGPE by changing scan rates (0.1−0.2 V/s). (**b**) Plot of I_pa_ versus ʋ^1/2^. (**c**) CVs obtained for PGAMGPE in the same electrolytic solution at different scan rates from 0.1 to 0.2 V/s. (**d**) Plot of I_pa_ versus ʋ^1/2^.

Using the conventional redox probe, the CVs were carefully examined at a range of scan rates from 0.1 V/s to 0.2 V/s. Figure 3a,c shows that, along with the linear increase in the redox peak current, the E_pa_ and E_pc_ shift to further positive and further negative sides, respectively. The plot of I_pa_ versus ʋ^1/2^ (Figure 3b,d) expresses linearity with R^2^ value of 0.9920 and 0.9932 for the BGPE and the PGAMGPE, respectively. The electroactive surface area of the BGPE and the PGAMGPE were calculated using Randles–Sevcik equation:

I_pa_ = 2.69 × 10^5^ n^3/2^ A C_o_ D^1/2^ ʋ^1/2^ (1)

I_pa_ is the oxidation peak current (µA), n represents the number of electrons exchanged, C_o_ represents the concentration of electroactive species, D for diffusion coefficient, and ʋ is the scan rate. The active surface area ‘A’ was determined using the slope of the plot of I_pa_ versus ʋ^1/2^. For the PGAMGPE, the electroactive surface area was higher (0.0 62 cm^2^) as compared with the BGPE (0.040 cm^2^).

The electron transfer rate constant (*k*^0^) value of the proposed electrode was calculated from the R_ct_ value using Equation (2):

(2) $$k^{0}=\frac{RT}{n^{2}F^{2}AC_{o}R_{ct}}$$

where *C_0_* is the concentration of the redox probe, *R* stands for the gas constant (8.314 J/K mol), *T* stands for the temperature in *K*, and *F* stands for Faraday’s constant (96,485 C/mol). The calculated value of *k*^0^ was found to be 1.63 × 10^−1^ cm/s.

### 3.3. Electrochemical Deposition of PGA Film on the Surface of the BGPE

**Figure 4:**
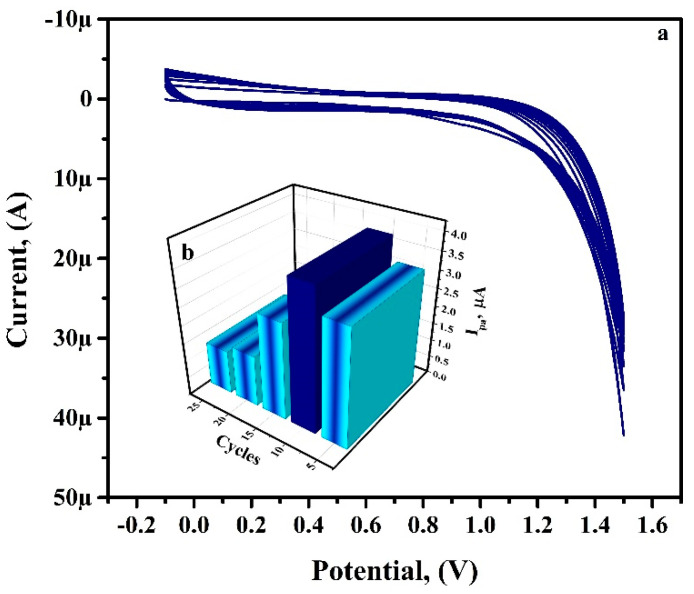
(**a**) CVs of electrochemical polymerisation of GA (0.1 mM) in 0.2 M PB of pH 6.5 in the potential window −0.1 V to 1.5 V at ʋ = 0.1 V/s. (**b**) Plot of the I_pa_ of 0.1 mM GA versus the number of polymerisation cycles.

**Scheme 1:**
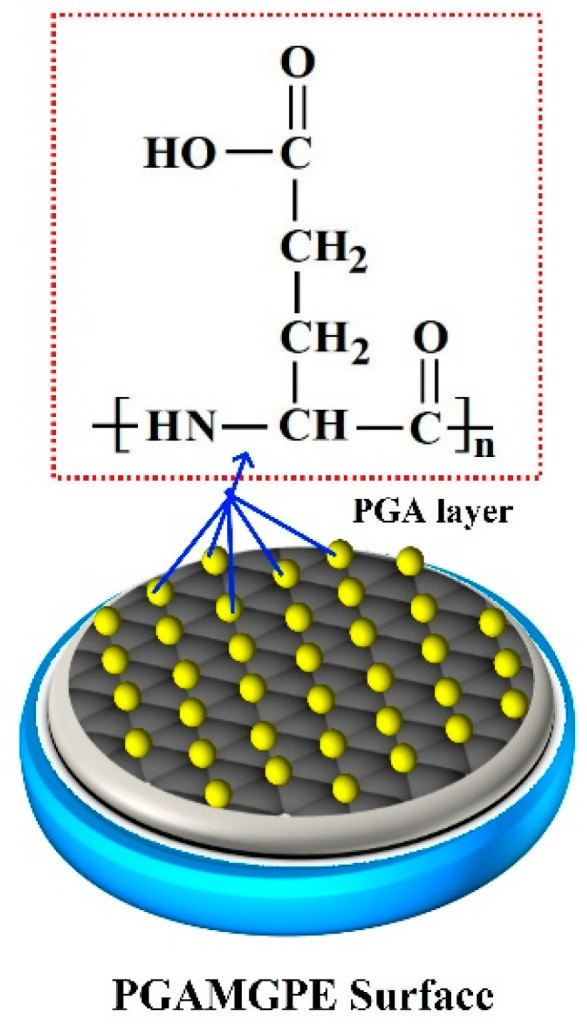
Possible electrochemical polymerisation of GA at the graphene surface.

A polymer coating was formed on the surface of the BGPE, by electropolymerising 1.0 mM GA in PB of 5.5 pH. Figure 4a illustrates the CV measurements made by continuously running 10 CV cycles in the potentials between −0.1 and 1.5 V at a scan rate of 0.1 V/s in a three-electrode electrochemical cell. Steadily improved CVs with the increased cycling time revealed the change of monomer GA into PGA on the surface of the BGPE. From Scheme 1, it is obvious that PGA is formed by the repetitive polymeric linkages between the α-amino and the δ-carboxylic acid groups [24]. Scan cycles were optimised by varying it from 5 to 25 cycles to obtain a PGA film of various thickness on the surface of the BGPE. Figure 4b indicates that at the 10th cycle enhanced current sensitivity was observed since complete surface coverage of the BGPE takes place at 10th cycle which marks the increase of active sites, and after that, the electron transporting tendency of PGA gets depleted due to which the peak current gets diminished. As an outcome, it was decided that 10 scan cycles would be ideal for further investigation.

### 3.4. Influence of Supporting Electrolyte

**Figure 5:**
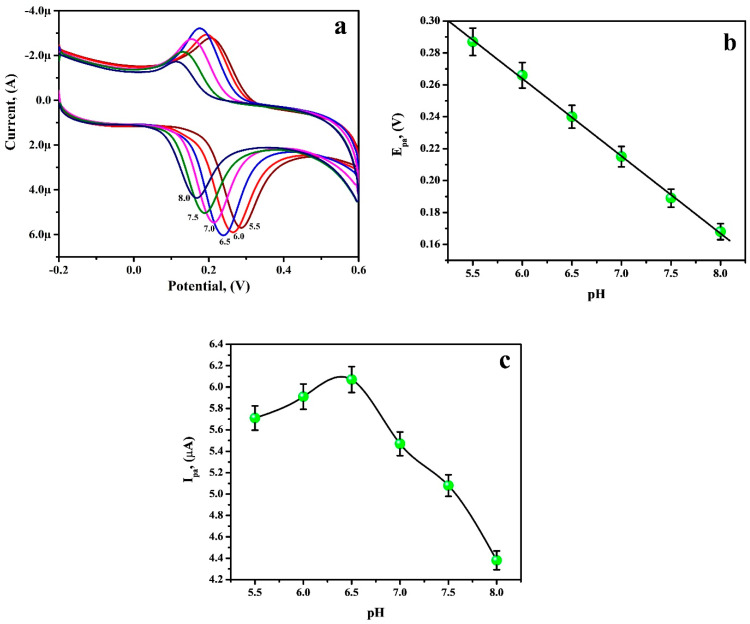
(**a**) CV response of 0.1 mM RU at PGAMGPE in 0.2 M PB of pH ranging from 5.5 to 8.0. (**b**) Plot of E_pa_ versus Ph. (**c**) Plot of I_pa_ versus pH of solution.

**Scheme 2:**
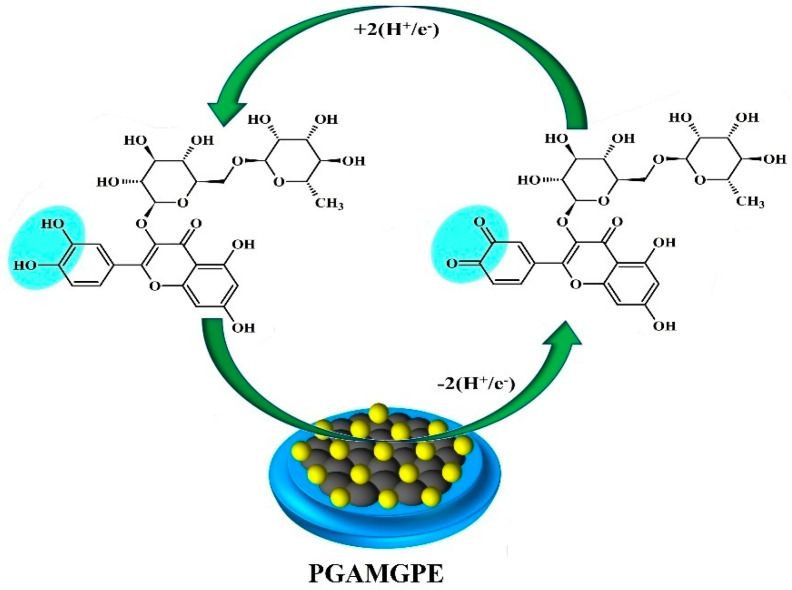
Possible electrochemical redox reaction of RU at the surface of PGAMGPE.

The electrochemical behaviour of RU on the PGAMGPE is significantly influenced by the buffer’s pH. It is possible to achieve a sharper response accompanied by increased sensitivity by optimizing the pH of a solution. Therefore, by using the CV technique, the effect of pH on the redox potential and current in 0.2 M PB at different pH levels extending from 5.5 to 8 was evaluated. The cyclic voltammogram generated by changing the pH from 5.5 to 8.0 is as shown in Figure 5a. The linearity equation E_pa_ [V] = 0.5550 – 0.0500 [V/pH] with R^2^ = 0.9994 represents the linear relationship between E_pa_ and pH (Figure 5b). It is clear from Figure 5b that as the pH rises, the peak potential shifts to the less positive side. The slope value of 0.050 V/pH is very near to the expected value of 0.059 V/pH, indicating that there are exactly the same number of proton and electron transfers occurring during the electrochemical oxidation of RU at the PGAMGPE. The CV outcome demonstrates that ΔI_p_ value rises with rising pH, reaches an enhancement at 6.5, and then falls. Based on the plot ‘I_pa_ versus pH’ (Figure 5c) pH 6.5 shows an enhanced peak current, and hence, it was considered to be the optimal condition for further analysis. Peak current magnitude is related to the rate of the electrochemical redox process. The probable mechanism is represented in Scheme 2. The number of protons involved in the electrochemical redox reaction of RU was found out by the Nernst Equation (3) [25]:

(3) $$B=\frac{-2.303\;mRT}{nF}$$

where *B* defines the slope of plot E_pa_ versus pH, m: the number of participated protons, and terms such as *R*, *T*, and *F* gas constant, experimental temperature and Faraday constant have their own physical significant values. The computed value of the proton count was determined to be 1.72, almost equivalent to 2.

### 3.5. Optimisation of Accumulation Potential and Time

**Figure 6:**
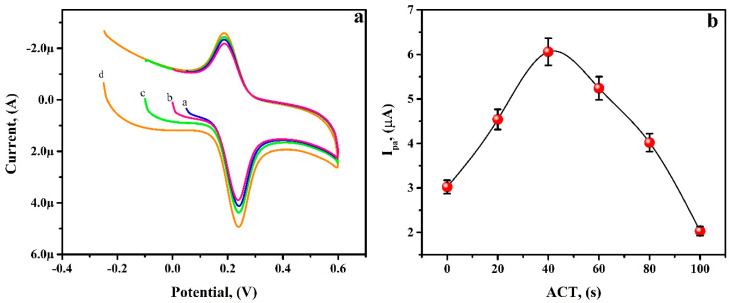
(**a**) CVs of accumulation potential versus I_pa_. (**b**) ACT versus I_pa_ in optimum pH at a scan rate of 0.1 V/s.

The accumulation time and potential are the vibrant parameters to enhance the highest adsorption of RU at the surface of PGAMGPE to attain a fine electrochemical reaction with low LOD and great sensitivity [26]. Figure 6a indicates an amplified redox peak current in the potential scale of −0.250 to 0.60 V, signifying that at this accumulation potential window utmost adsorption of RU at the PGAMGPE surface occurs. Hence, for the investigation of RU, the potential gap of −0.250 to 0.60 V was chosen as the best accumulation potential gap. Figure 6b shows the plot of time versus I_pa_, which unveils that from the obtained electrochemical results at different values of ACT from 0.0 to 100.0 s, the maximum I_pa_ of RU was attained at 40.0 s. The decrease in RU current that possibly occurred due to the electrode surface getting saturated due to the full surface coverage of RU at 40 s of ACT. Therefore, the value 40 s was selected as the better accumulation time for the analysis RU.

### 3.6. Electrochemical Sensing of RU at the PGAMGPE

**Figure 7:**
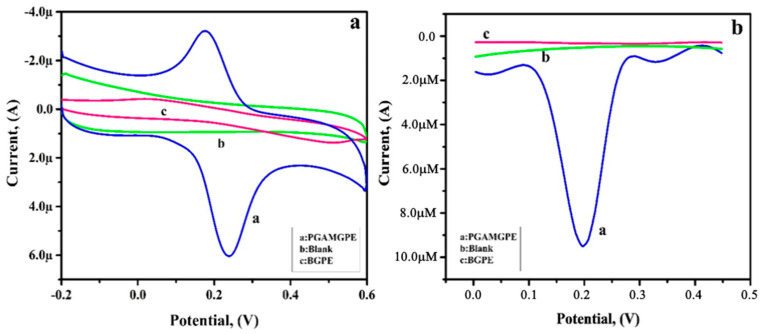
(**a**) CV response of 0.1 mM RU at PGAMGPE (curve a), BGPE (curve c), and blank (curve b) with the scan rate of 0.10 V/s. (**b**) DPVs for 0.1 mM RU on PGAMGPE (curve a), at BGPE (curve c), and blank (curve b).

Figure 7a demonstrates that the response of CV for 0.1 mM RU displays a less noticeable electrochemical redox peak at the BGPE (curve c), signifying that the adsorption of RU at the BGPE is feebler with a less sensitive electrochemical reaction. It can, however, produce two peaks at the PGAMGPE (curve a) electrode surface in 0.2 M PB of pH 6.5 with the I_pa_ and I_pc_ of 5.37µA and −3.45 µA, respectively. The E_pa_ and E_pc_ are 0.240 V and 0.180 V, with a peak separation of about 0.060 V and the I_pa_/I_pc_ ratio of 1.55, demonstrating electrochemical reversibility for RU at the PGAMGPE. This should be credited to the accumulation and promotion effect of PGA which increases the rate of electron transfer. Similarly, during the absence of RU molecule (curve c) in PB at the PGAMGPE, we did not notice any of the redox peak. The results of differential pulse voltammograms (DPVs) (Figure 7b) parade the electrochemical activity of RU on the surfaces of the PGAMGPE (curve a) and the BGPE (curve c) and of the blank solution. At the PGAMGPE, an enhanced peak current is observed in comparison to the BGPE for RU. Likewise, the DPV response for only 0.2 M PB solution does not display any peak current (curve b).

The surface concentration ‘Г’ of RU on the PGAMGPE surface was found to be 1.7 ÅM/cm^2^ based on the following equation [27]:

Q = nFAΓ(4)

Here, n is the number of electrons involved, and Q is the charge from the area of the CV peak under ideal conditions. 

### 3.7. Potential Scan Rate Effect

**Figure 8:**
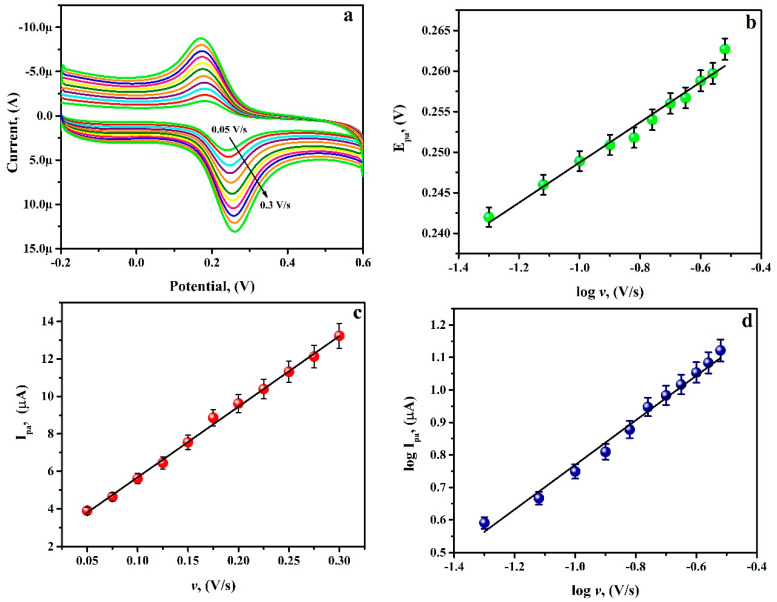
(**a**) CVs for 0.1 mM RU at PGAMGPE in pH 6.5 at different scan rates ranging from 0.050 to 0.30 V/s. (**b**) Graph of E_pa_ versus log ʋ. (**c**) Graph of I_pa_ versus ʋ. (**d**) Graph of log I_pa_ versus log ʋ.

The scan rate effect on the electrochemical peak current of RU in pH 6.5 of PB at the PGAMGPE was studied. From Figure 8a, it is obvious that a distinct reversible redox peak was attained and the CVs gradually increased with the scan rate from 0.050 to 0.30 V/s, and when the potential scan rate rises, the positive shif of E_pa_ and the negative shift of E_pc_ with the increase in the peak separation was observed. This shows that the rate of electron transfer is not relatively fast and that the reversibility of the electrochemical reaction eventually decreases [28]. The plot of log ʋ against E_pa_ shows good linearity with the equation as follows (Figure 8b):

E_pa_[V] = 0.2736 + 0.0249 log ʋ, (V/s) (R^2^ = 0.9989) (5)

Furthermore, the plot of I_pa_ against ʋ (Figure 8c) and the plot of log I_pa_ against log ʋ (Figure 8d) shows a good linear correlation and the corresponding linear regression equations are:

I_pa_ (μA) = 37.65 + 1.9185 ʋ (V/s) (R^2^ = 0.9954) (6)

log I_pa_ (µA) = 1.4754 + 0.9989 log ʋ, (V/s) (R^2^ = 0.9980) (7)

which specifies that the electrochemical reaction process of RU at the PGAMGPE surface is controlled by an adsorption pathway. The slope of Equation (7) is in close proximity with the hypothetical value of one for the adsorption-controlled reaction pathway [29]. Considering the slope of I_pa_ versus scan rate, Equation (8) estimates that there were two electrons transported, which indicates a two-electron and two-proton mechanism for RU [30].

(8) $$Ipa=\frac{n^{2}F^{2}vA\Gamma }{4RT}=\frac{nFQv}{4RT}$$

The charge transfer coefficient (α) was premeditated by the Bard and Faulkner relation (Equation (9)) and was obtained to be 0.36:

(9) αn=47.7Epa−Epa2

*E_pa_*_/2_ represents the half wave anodic potential, and *E_pa_* is the anodic potential under the optimised condition at 0.1 V/s scan rate.

### 3.8. Analysing Capability of PGAMGPE towards RU

**Figure 9:**
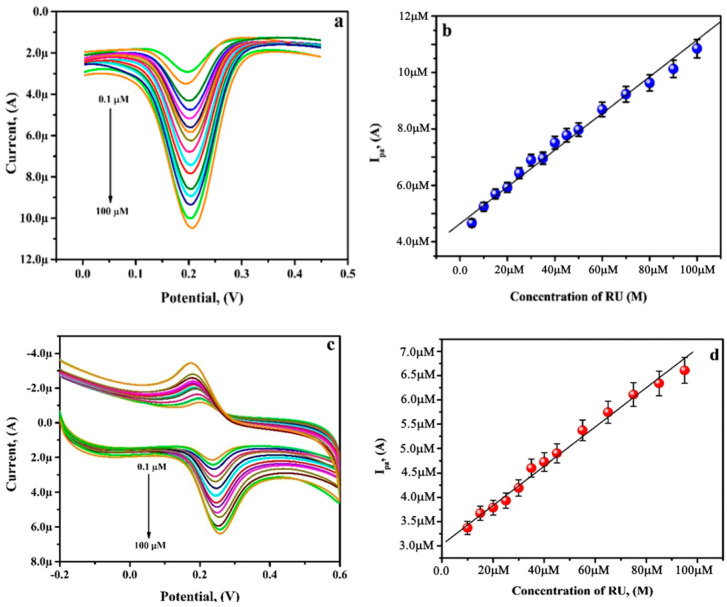
(**a**) DPVs for varying concentrations of RU ranging from 0.10 to 100.0 μM in PB on PGAMGPE. (**b**) Plot of [RU] versus I_pa_. (**c**) CVs for varying RU concentrations ranging from 0.10 to 100.0 μM, in PB at PGAMGPE. (**d**) Plot of [RU] versus I_pa_.

The BGPE and the PGAMGPE’s electrocatalytic capabilities were examined using the CV and DPV procedures under ideal conditions. In the DPV and CV voltammograms shown in Figure 9a,c, an increase in the peak current is observed concurrently with an increase in concentration from 0.1 µM to 100 µM. The calibration plot of DPV (Figure 9b) and CV (Figure 9d) displays that the RU oxidation peak current at the PGAMGPE has good linear proportionality to its concentration over the range of 0.10 to 100.0 µM in PB. The linearity fitted results are as follows:

I_pa_ (µA) = 4.6429 × 10^−6^ + 0.6652 [RU](M), (R^2^ = 0.9930) by DPV method

I_pa_ (µA) = 3.0283 × 10^−6^ + 0.4403 [RU] (M), (R^2^ = 0.9947) by CV method

The LOD and limit of quantification (LOQ) at the surface of the PGAMGE were assessed by considering the slope values (B) of the calibration plot and standard deviation (S) of the blank solution in the following relations [31]:

(10) $$LOD=\frac{3S}{N}$$

(11) $$LOQ=\frac{10S}{N}$$

The premeditated *LOD* and *LOQ* were achieved to be 0.04 µM and 0.15 µM using the DPV method and 0.06 µM and 0.21 µM using the CV method, respectively. The sensitivity of the modified electrode was found to be 10.72 A/M/cm^2^, which was calculated by the equation

(12) $$Sensitivity=\frac{B}{A}$$

A is the electrochemical surface area of the modified electrode and B is the slope of the calibration plot. The results given for the analytical parameters of RU at the modified surface of various electrodes are identical to the analytical parameters attained at the PGAMGPE, as shown in Table 1 [32,33,34,35,36,37,38,39,40]. Additionally, the PGAMGPE results validate that the PGAMGPE is an appropriate tool for the sensitive electrochemical detection of RU with a significant improvement in the simplified fabrication process.

### 3.9. Simultaneous Analysis of RU and CF

RU is a bioflavonoid naturally found in certain foods such as apple peels, green tea, black tea, onions, and many citrus fruits. In addition to acquiring it from food, RU is also available as a supplement in the form of pills or capsules. The phytochemical nature of RU offers pharmacological benefits for the treatment of a number of chronic diseases, including diabetes, hypertension, high blood cholesterol, and cancer. As a result, RU has a wide range of positive impacts on human health. CF is also an alkaloid extensively found in natural products used in beverages. Numerous physiological effects include diuresis, activation of the central nervous system, and stomach acid secretion. CF combined with nonsteroidal anti-inflammatory medicines in analgesic formulations is therapeutically used to alleviate migraines [41]. So, it has been suggested that RU and CF may have similar in vivo effects [42]. Hardly any methods have been tested for the concurrent analysis of RU and CF. Hence, the fabricated sensor is used for the concurrent determination of RU and CF.

**Figure 10:**
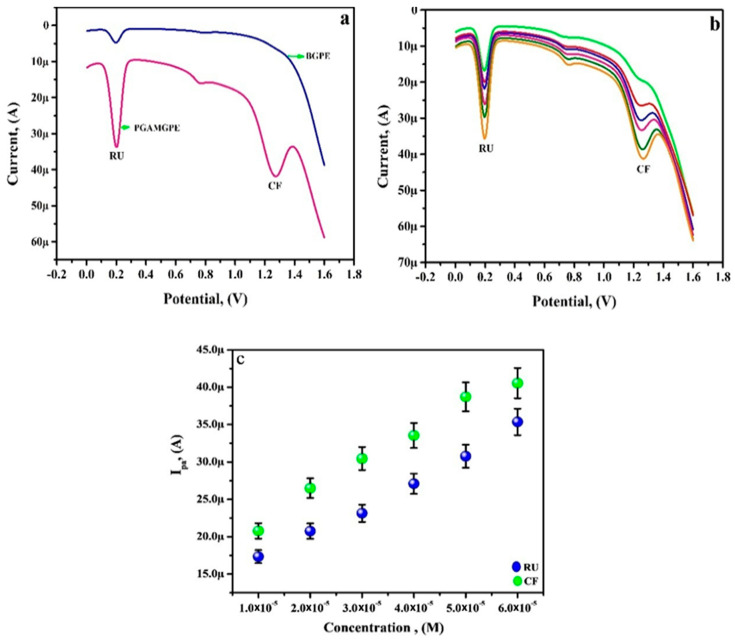
(**a**) DPVs for simultaneous analysis of RU, CF (0.1 mM) at PGAMGPE and BGPE in PB. (**b**) DPVs for simultaneous investigation with variable concentrations (10.0 to 60.0 µM) of RU and CF. (**c**) Plot of I_pa_ versus [RU] and [CF] under optimum condition.

Using the PGAMGPE, the simultaneous measurement of 0.1 mM solutions of RU and CF was accomplished in PB at 0.1 V/s scan rate. Figure 10a displays the DPV for concurrent analysis of RU and CF at the PGAMGPE and at the BGPE. The DPV for the simultaneous determination of RU and CF at the PGAMGPE and at the BGPE is shown in Figure 10a. For RU and CF, the PGAMGPE exhibits two well-separated peaks with higher current responsiveness at peak potentials of 0.2014 V and 1.2580 V. The DPV obtained by gradually increasing the concentration of RU and CF from 10 µM to 60 µM under optimal conditions is shown in Figure 10b. The voltammograms produced demonstrate that the peak current increases as concentration increases. The plot of I_pa_ versus [RU] and [CF] (Figure 10c) displays linearity with correlation coefficients of 0.9920 and 0.9921. RU and CF can, therefore, be determined simultaneously using the PGAMGPE with a favourable performance.

### 3.10. Interference Analysis

The choosiness of the modified electrodes will be severely influenced by the presence of certain inactive substances in pharmaceutical samples that are formed alongside the active ingredient of the prescription drug. Under ideal conditions and with the presence of concentrated solutions of some foreign substances such as glycine, caffeine, glucose, and urea, DPV analysis of RU (0.1 mM) at the PGAMGPE was performed. Biologically present cations such as Na^+^, Mg^2+^, K^+^, and Ca^2+^ were added to the modified sensor in order to study its selectivity. The observations demonstrated that the presence of possible interferents has no influence on the redox potential of RU and that the PGAMGPE may be utilised to accurately determine RU.

### 3.11. Assessment of Stability, Reproducibility, and Repeatability

The stability of the PGAMGPE was analysed by verifying CVs of 60 segments for RU in PB. The degradation percent of the current response was premeditated by calculating the preliminary (I_pa (1)_) and final currents (I_pa (n)_) which was found to be 98%. Since 98% of the original current was continued even after 30 cycles, the constructed sensor was found to be stable. The reproducibility of the electrode was recorded by running five consecutive CV cycles for RU in PB at four independently modified PGAMGPEs. The attained RSD value of 4.51% conveys that the PGAMGPE is reproducible with high accuracy. Five successive CVs with the same modified electrode yielded a stable, repeatable peak that had an RSD of 3.89% for four trials, signifying that the PGAMGPE may be operated for multiple measurements.

### 3.12. Analytical Application of PGAMGPE

**Figure 11:**
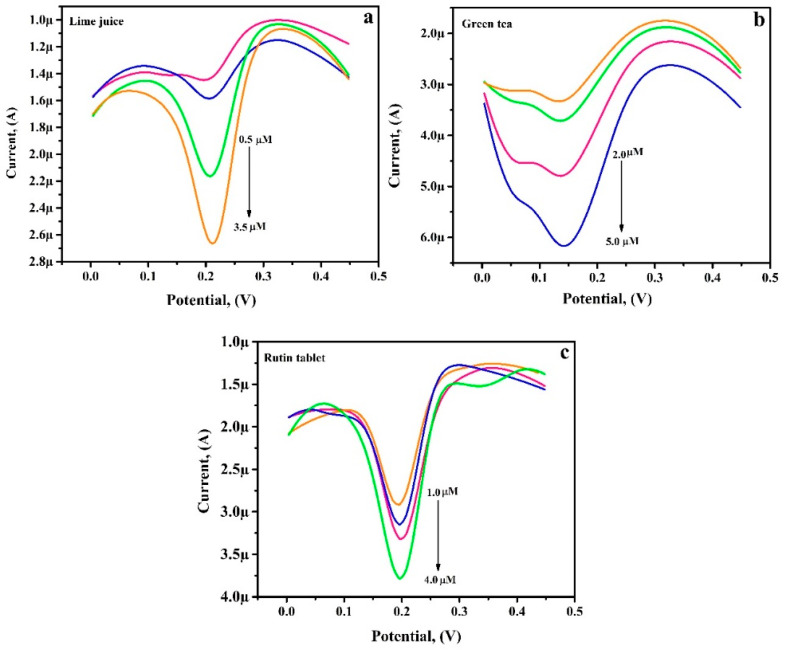
DPVs for RU detection in (**a**) lime juice, (**b**) green tea, and (**c**) tablet samples at PGAMGPE.

The analytical utility of the PGAMGPE for the examination of RU was verified in samples such as lime juice, green tea, and tablet samples by the DPV technique. According to the analytical DPV responses for lime juice from 0.5 to 3.5 µM (Figure 11a), green tea from 2.0 to 5.0 µM (Figure 11b), and RU tablets from 1.0 to 4.0 µM (Figure 11c) under optimised conditions, the peak current rises as the concentration of the real samples does. The RU real sample solutions were analysed by diluting them with 0.2 M PB of pH 6.5 with concentrations ranging from 10 to 40 µM. Since, initially, there were no electrochemical peaks for RU in the diluted lime juice sample, the standard addition procedure was used. However, RU-containing green tea and tablet samples display the electrochemical reaction for RU without adding a commercial sample of RU. Because of the excellent recovery, the modified electrode (PGAMGPE) is appropriate for all real sample studies. The outcomes are listed in Table 2.

## 4. Conclusions

In the current research work, the PGAMGPE was effectively used to examine the electrochemical redox reaction nature of RU. FE-SEM, EIS, and CV techniques were used to successfully characterise the fabricated sensor materials. The PGAMGPE displays a high catalytic nature in comparison with the BGPE for electrochemical redox reaction of RU with an improved current sensitivity using CV and DPV methods. The most efficient redox reaction process was obtained at pH 6.5, demonstrating that it is adsorption-controlled and includes two electrons and two protons. The impact of accumulation potential and time on electrochemical behaviour of RU at the PGAMGPE was deliberated effectively. The PGAMGPE achieves an acceptable linear range for varying RU concentrations with a low LOD of 0.04 µM and 0.06 µM by DPV and CV approaches, respectively. Additionally, the PGAMGPE provided remarkable recovery for RU in lime juice, green tea, and tablet samples. The PGAMGPE can become the utmost relevant sensing device for the examination of RU on account of its remarkable electrocatalytic activity and certain advantageous properties including great analysing efficiency, high selectivity, sensitivity, repeatability, reproducibility, and stability.

## Figures and Tables

**Table 1:** Comparison of the present RU LOD at PGAMGPE with the previous RU sensors.

Technique	Electrode	Linear Range (µM)	LOD (µM)	Reference
SWV ^a^	CPE ^b^	1–100	0.850	[32]
SWV	α-C16-GCE-MWCNT ^c^	2–8	0.604	[33]
DPV	Mg-Al-Si-PC-GCE ^d^	1–10	0.01	[34]
DPV	MWCNTs-IL-gel-GCE ^e^	0.04–100	0.01	[35]
DPV	Co-ZIF-C-GCE ^f^	0.1–30	0.022	[36]
DPV	CB-WO_3_-SPCE ^g^	0.01–75.46	0.002	[37]
CV	SPE ^h^	2.0–15	0.1	[38]
CV	CMB-GNPs-CBCPE ^i^	0.1–8	0.023	[39]
HPLC-UV	^/^	16.3–98.27	3.0	[40]
CV	PGAMGPE	0.1–100	0.06	Present
DPV	PGAMGPE	0.1–100	0.04	work

^a^ Square wave voltammetry; ^b^ Carbon paste electrode; ^c^ α-Carbohydrate-doped multiwalled carbon nanotube modified glassy carbon electrode; ^d^ Porous carbon encapsulated Mg-Al-Si alloy nanocluster; ^e^ Ferrocene benzyne derivative immobilised gold nanoparticle modified glassy carbon electrode; ^f^ Magnetic micromesoporous graphitized carbon wrapped Co nanoarchitecture; ^g^ WO_3_-infused carbon based nanohybrids; ^h^ Screen-printed disposable carbon paste electrode; ^i^ Cetrimonium bromide graphene nanoplatelets carbon paste composite electrode.

**Table 2:** Recovery of RU in samples.

Sample	Added (µM)	Found (µM)	Recovery (%)
Lime Juice	0.5	0.48	96.83
	1.0	0.97	97.67
	2.0	1.90	95.00
	3.0	2.83	97.88
Green Tea	2.0	1.89	94.64
	3.0	2.91	97.13
	4.0	4.10	102.68
	5.0	4.95	99.04
RU Tablet	1.0	0.99	99.89
	2.0	2.06	100.31
	3.0	2.95	98.40
	4.0	3.98	99.74

## Data Availability

The data underlying this article are presented in the main manuscript. The datasets generated during and/or analysed during the current study are available from the corresponding author upon reasonable request.
